# Sex and age modulate antennal chemosensory-related genes linked to the onset of host seeking in the yellow-fever mosquito, *Aedes aegypti*

**DOI:** 10.1038/s41598-018-36550-6

**Published:** 2019-01-10

**Authors:** Anaïs Karine Tallon, Sharon Rose Hill, Rickard Ignell

**Affiliations:** Disease Vector Group, Department of Plant Protection Biology, Swedish University of Agricultural Sciences, Alnarp, Sweden

## Abstract

The mosquito *Aedes aegypti* is the primary vector for the fastest growing infectious disease in the world, dengue fever. Disease transmission heavily relies on the ability of female mosquitoes to locate their human hosts. Additionally, males may be found in close proximity to humans, where they can find mates. Host seeking behaviour of both sexes is dependent on adult sexual maturation. Identifying the molecular basis for the onset of host seeking may help to determine targets for future vector control. In this study, we investigate modulation of the host seeking behaviour and the transcript abundance of the main chemoreceptor families between sexes and across ages in newly-emerged mosquitoes. Attraction to human odour was assessed using a Y-tube olfactometer, demonstrating that both males and females display age-dependent regulation of host seeking. The largest increase in transcript abundance was identified for select chemosensory genes in the antennae of young adult *Ae*. *aegypti* mosquitoes and reflects the increase in attraction to human odour observed between 1 and 3 day(s) post-emergence in both males and females. Future functional characterisation of the identified differentially abundant genes may provide targets for the development of novel control strategies against vector borne diseases.

## Introduction

The yellow fever mosquito, *Aedes aegypti*, is the primary vector of dengue, yellow fever, chikungunya and Zika^1^. The rapid and ongoing geographic expansion of the vector and its concordant diseases strongly reflects the infection burden in affected regions, and directly correlates with the biology of *Ae*. *aegypti*^2,3^. A key determining factor for disease transmission by mosquitoes is host seeking, which relies on the capacity of female mosquitoes to locate a human host and successfully blood feed^3–5^. A better understanding of host seeking, and its molecular mechanisms and regulation, is likely to lead to the identification of novel targets and strategies against vector borne diseases.

Host seeking in mosquitoes relies heavily on olfaction to detect and integrate ecologically relevant cues^4,5^. The primary olfactory appendage, the antenna, is covered by hair-like sensilla housing the odorant sensory neurons (OSNs) and support cells, which express an array of chemosensory proteins that play an integral role in odour detection and recognition^6^. Odorant binding proteins (OBPs) are small globular proteins, secreted by the support cells, and which constitute the main chemosensory protein family present in the sensillum lymph^6^, with 35 and 36 genes identified as expressed in the antennae of male and female *Ae*. *aegypti*, respectively^7,8^. These proteins are putatively involved in ligand binding and transport, as well as in gain control^6,9^. The chemosensory proteins (CSPs) play a role similar to OBPs^10,11^, with 12 and 17 found to be expressed in antennae of male and females *Ae*. *aegypti*, respectively^7^.

Detection of odorants in the antenna of insects is dependent on the expression of several chemoreceptor families in the dendritic membrane of the OSNs, including odorant receptors (ORs), ionotropic receptors (IRs) and gustatory receptors (GRs)^6^. The ORs are seven-transmembrane domain proteins, which form heteromeric receptors consisting of a highly conserved co-receptor, ORco, and a rapidly evolving variable tuning OR that determines the selectivity and sensitivity of the receptor complex, and thus the OSNs^12,13^. A total of 75 and 84 OR genes have been identified to be expressed in male and female *Ae*. *aegypti* antennae, respectively^7,14^. In contrast to the ORs, the antennal IRs are a family of highly conserved ligand-gated ion channels^15^. A total of three IR co-receptors, IR25a, IR8a or IR76b, and 34 and 41 tuning IRs have been described as expressed in male and female *Ae*. *aegypti*, respectively^7^. These heteromeric receptors have been shown to play roles as chemosensory receptors in *Anopheles coluzzii*^16^. While GRs have been shown to play a role in the detection of CO_2_ in the maxillary palps of mosquitoes^17–19^, the functional role of the 27 and 29 GRs expressed in the antennae of females and males, respectively^7^, is unknown.

Besides members of the canonical chemoreceptor families, low numbers of pickpocket (PPK)^20^ and transient receptor potential (TRP) ion channel genes^21^ [and references therein] have been described as expressed in the antennae of both sexes of *Ae*. *aegypti*^7^. Although not all PPKs and TRPs are chemosensory, recent studies in *Drosophila melanogaster* have ascribed such a role to a subset of these receptors^22^ [and references therein]. A total of 43 and 51 PPK and TRP genes, including homologs of the *D*. *melanogaster* chemosensory receptors, have been found to be expressed in the antennae of male and female *Ae*. *aegypti*, respectively^7^. Moreover, a family of membrane bound proteins known to be associated with the chemoreceptors is the sensory neuron membrane proteins (SNMPs), SNMP1 and SNMP2^23,24^, both of which are expressed in the antennae of *Ae*. *aegypti*^7^. While SNMP1 has been shown to be involved in pheromone detection in other insects^23^, its functional role in mosquitoes remains unknown.

Expression of host seeking in mosquitoes is dependent on sex, age and physiological state^25–30^. Female mosquitoes gradually develop their behavioural and physiological competence to seek and feed on human blood after adult emergence in order to complete their gonotrophic cycle^29,31,32^. Following a successful blood meal, females are refractory to host odour until after oviposition^27,28,33^. These changes in behaviour are correlated with differential changes in chemosensory gene transcript abundance [Omondi *et al*.^7,25,26,34,35^ submitted; Hill *et al*. submitted] and in the sensitivity of the OSNs tuned to salient human odorants, as shown for *Ae*. *aegypti* and *An*. *coluzzii*^25,26,31^. Even though functional genomic analyses in *Ae*. *aegypti* have revealed important contributions of select antennally expressed chemosensory receptors to host attraction and discrimination^36,37^, the functional significance of individual tuning ORs and IRs is largely unknown. In *An*. *coluzzii*, on the other hand, recent studies have revealed a subset of chemosensory receptors to be involved in the detection of salient human odorants [Omondi *et al*.^17,35^; submitted]. The studies by Omondi *et al*. also provide evidence of a direct functional correlation between the expression of the chemosensory genes encoding for these receptors, the sensitivity of the OSN responding to their cognate ligands, and behavioural expression. Whether the display of host seeking in female *Ae*. *aegypti* is regulated through a similar mechanism is yet unknown.

Host seeking in *Ae*. *aegypti* is not exclusive to females, as males are attracted to human odour and are found in close association with their hosts for mate location^30,38,39^. Interestingly, this behaviour is also age dependent, with one day post-emergence (dpe) males being indifferent to human odours^30,39^. Thus, both sexes display host seeking related to reproductive behaviour at times corresponding to their ecological needs. Yet, there is a clear sexual dimorphism in the number of antennal sensilla, with males having approximately one third of that of females^40^, and in the expression of select chemosensory genes^7,14^. As of yet, no studies have analysed the molecular machinery involved in regulating the odour-mediated behaviours of male mosquitoes. Such studies may be pertinent for the understanding of the behavioural ecology, and the role in vectorial capacity, of this often neglected sex.

In this study, we investigate the effect of sex and age on the transcript abundance of the main chemosensory gene families, through quantitative mRNA sequencing (RNA-seq), and correlate this with the behavioural responses of male and female *Ae*. *aegypti* to human odours, from one day to five dpe. The observed differential behavioural changes in both males and females to human odours with age is reflected in an overall qualitative and quantitative difference in antennal chemosensory transcript abundance. We discuss our findings in relation to the current understanding of the functional characteristics of the chemosensory system in mosquitoes.

## Results and Discussion

### Behaviour

**Figure 1:**
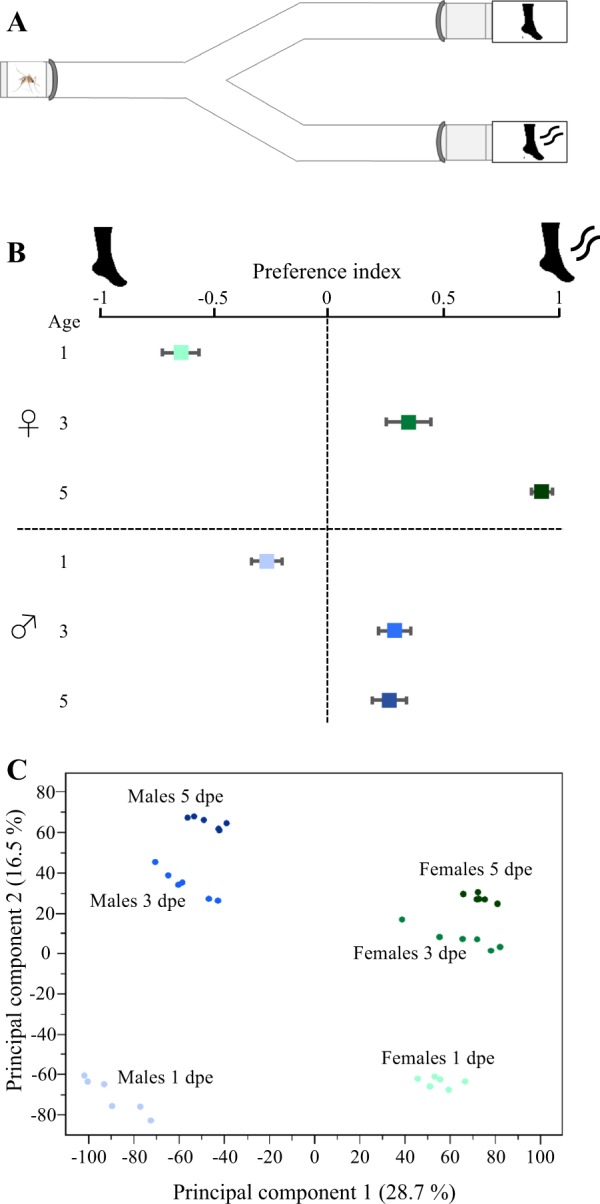
*Aedes aegypti* responds to human odour sex- and age-dependently, reflected in overall antennal transcript abundance. (**A**) Diagram of the Y-tube olfactometer used to assess the behavioural preference of male and female mosquitoes to human odour. (**B**) Behavioural analysis of females and males, scored as preference indices, revealed both sex- and age-dependent responses to human odour. Age is defined as the number of days post-emergence. Error bars represent standard error. (**C**) A principal component analysis representing the overall transcript abundance in the antennae of female and male *Ae*. *aegypti*, at 1, 3 and 5 days post-emergence (dpe). The two major principal components explain for 45.2% of the variance for the six biological replicates of each sex and age, as indicated by different colours. All diagrammatic representations are courtesy of Pixabay, an open source image database.

Females and males displayed a sex- (χ^2^ = 13.03, df = 1, *P* = 0.0111) and age-dependent (χ^2^ = 42.86, df = 1, *P* < 0.001) behavioural response to human odour when assessed in a Y-tube olfactometer (Fig. 1A). Females exhibited a stark change in behavioural response between 1 and 3 dpe (*P* = 0.0037; Fig. 1B), as well as between 1 and 5 dpe (*P* = 0.0002; Fig. 1B). The 1 dpe females demonstrated a preference for the control (*P* = 0.016), while older females preferred the arm containing human odours (*P* < 0.001). No significant difference in preference was observed between 3 and 5 dpe females (*P* = 0.087; Fig. 1B). The observed avoidance of human odour in newly-emerged females is in line with previous studies on *An*. *coluzzii* (Omondi *et al*., submitted). The gradual age-dependent onset of attraction has been observed in both *An*. *coluzzii* [35; Omondi *et al*., submitted] and *Ae*. *aegypti*^26,31^, in which ca. 90% of females *Ae*. *aegypti* are competent to host seek between 3 and 5 days following adult-emergence. The ecological relevance of the observed age-dependent attraction in mosquitoes likely relates to balancing the risk of approaching a human with the need for obtaining a mate and a blood meal for egg development^29,30,32,38,39^. Although males did not exhibit attraction to or avoidance of human odour at all ages (Fig. 1B), we observed a change in the behavioural response between 1 and 3 dpe (*P* = 0.0227) and 1 and 5 dpe (*P* = 0.0259; Fig. 1B). Similar observations have been made by Paixão *et al*.^30^, in which males older than 3 dpe exhibited an increased flight behaviour towards human odour. This vertebrate host odour attraction is directly linked to swarming in sexually mature males^38,39^. Male *Ae*. *aegypti* do not engage into sexual behaviours such as swarming and copulation prior 24 h following adult emergence^39^. The observed similarity in host avoidance at 1 dpe implies that both males and females are constrained in risk taking. Alternatively, this behavioural response may be due to aging although this is unlikely as both sexes display similar level of activity independent of age.

### RNA sequencing

Quantitative single-end sequencing of antennal RNA from each of the 36 samples generated a mapping of over 22 million cleaned reads per library (Additional File 1). Out of the 18868 genes annotated in the genome of *Ae*. *aegypti*, we detected a total of 16008 transcripts in the antennae of both females and males, which is in line with the 15592 transcripts reported by Matthews *et al*.^7^. We report a total of 10065 reliably detected transcripts (Additional File 1), which are fewer than the 11388 transcripts previously reported by Matthews *et al*.^7^, likely reflecting the different age groups investigated in each study. The observed discrepancy, however, does not seem to relate to differences in sequencing depth, as the 100 least abundant genes identified in Matthews *et al*.^7^ were all reliably detected in this study.

To assess the depth and coverage of sequencing, the core eukaryotic gene mapping approach, common eukaryotic gene mapping approach (Cegma)^41^, was used^35^. A total of 405 transcripts of the 451 core eukaryotic genes identified in *Ae*. *aegypti* were reliably detected above the threshold of 1 RPKM (reads per kilobase of transcript length per million mapped reads), supporting a good coverage efficiency of our sequencing and a reliable estimation of gene expression.

### Overall and differential expression profiles

To assess the overall variation among the transcriptome libraries (6 replicates per sex and age group), specifically to identify and quantify any overall differences across sexes and age groups, a principal component analysis (PCA) of the antennal transcripts was conducted (Fig. 1C). All of the biological replicates of the same sex and age clustered tightly together in the principal component space, indicating that no significant differences were introduced into the libraries through handling and processing. The PCA revealed that 28.7% of the variation among the libraries was based on sex (PC1), while 16.5% of the variance was dependent on age (PC2) (Fig. 1C). The degree and direction of variation in the PC2 is interesting as it correlates with the observed differential host seeking behaviour in recently emerged mosquitoes (Fig. 1B).

Among all the genes (609) that were differentially detected in females, a total of 326 genes demonstrated lower abundance, while 283 were more abundant in 3 dpe when compared to 1 dpe mosquitoes (Additional File 2). Moreover, only 33 differentially detected genes exhibited lower abundance, while 18 were more abundant in 5 dpe compared to 3 dpe females (Additional File 2). Similarly for males, a total of 543 genes showed lower abundance among all the genes (1031) that were differentially detected, while 488 were more abundant in 3 dpe when compared to 1 dpe mosquitoes (Additional File 3). In males, only 76 differentially detected genes exhibited lower abundance, while 35 were more abundant in 5 dpe compared to 3 dpe mosquitoes (Additional File 3). It is interesting to note that only a small subset of genes are differentially detected during adult sexual maturation, with some likely involved in the regulation of onset of the host seeking behaviour.

**Figure 2:**
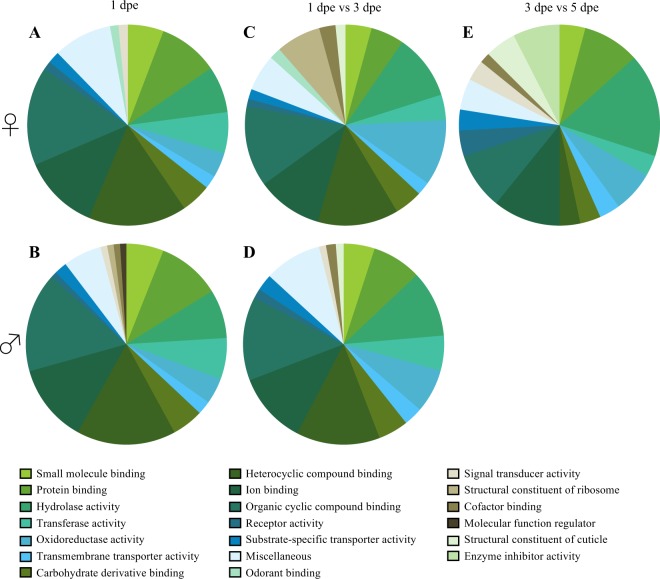
A level-three gene ontology analysis of molecular functions in the antennal tissue of *Aedes aegypti*. The proportion of genes that are reliably detected in 1 day post-emergence (dpe) females (**A**) and males (**B**), and the proportion of genes that are differentially abundant between 1 and 3 dpe females (**C**) and males (**D**). The proportion of functional classes differentially abundant between 3 and 5 dpe is only shown for females (**E**), as no differences were observed for males.

To further characterise which genes underlie the variation between sexes and among ages, a gene ontology (GO) analysis of molecular function (level three) was conducted. Among all the transcripts identified above the reliable level of detection (>1 RPKM) in both males and females, we observed a larger number of molecular functional classes in 1 dpe mosquitoes than older individuals (Fig. 2A,B). In contrast, the same number of classes were described for 3 and 5 dpe mosquitoes. The same molecular functional classes were found to be differentially detected between 1 and 3 dpe mosquitoes (Fig. 2C,D). The only exception to this was the functional class “odorant binding”, which was only present above the threshold of detection in females (Fig. 2A). A plausible explanation for this discrepancy is the reduced number of chemosensory sensilla present on male antennae^40^. Since the vast majority of chemosensory genes are included in the “odorant-binding” functional class, it is interesting to note that the shift in the number of these genes in females occurs between 1 and 3 dpe, consistent with the demonstrated onset of host seeking (Fig. 1B). Other functional classes found to be differentially detected with age in females are “structural constituent of ribosome”, “cofactor binding” and “molecular function regulator” (Fig. 2A–C,E). These functional classes are all involved in the modulation of the cellular activity, specifically translation and enzymatic activity, and the observed changes likely reflect the maturation of the more intricate chemosensory system in females. In addition, “structural constituent of cuticle” was differentially abundant between 3 and 5 dpe females (Fig. 2E). This differential abundance in the number of genes likely correlates with the delayed formation of the cuticular cortex of the flagellomere and the erection of antennal fibrilla in females^42^. As expected, the majority of changes in the number of transcribed genes occur among functional classes involved in the maturation of antennal structure and the chemosensory machinery in this complex olfactory tissue.

### Detection of chemosensory-related genes

**Figure 3:**
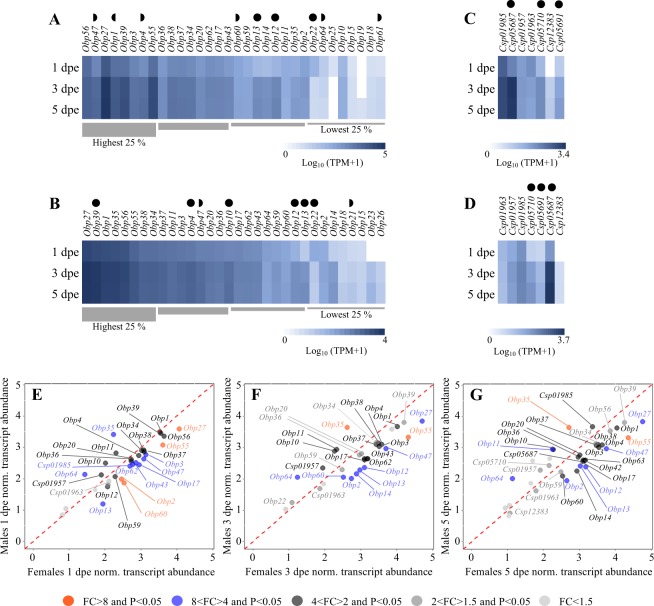
Antennal binding protein differential transcript abundance between sexes and across ages in *Aedes aegypti*. Heat plots showing the abundance of odorant binding protein (*Obp*) and chemosensory protein (*Csp*) transcripts in female (**A**,**C**) and male (**B**,**D**) *Ae*. *aegypti* at 1, 3 and 5 days post-emergence (dpe). Genes within each gene family are presented left to right from most to least abundant as determined by the mean abundance of all three ages for each sex. All *Obp* transcripts are ordered from left to right from the highest to the lowest quartile. Note differences in scale in (**A**–**D**). Genes differentially abundant between 1 dpe and 3 dpe individuals (◖), genes differentially abundant between 1 dpe and 5 dpe (◗), and genes differentially abundant between both 1 dpe and 3 dpe, and 1 dpe and 5 dpe (•), were identified using the beta-binomial Baggerley’s test and a false discovery rate (FDR)- corrected p-value (*P*) of <0.05 (n = 6) with a FC ≥2. Scatter plots showing the differential transcript abundance of *Obp*s and *Csp*s in the antennae of both sexes at 1 dpe (**E**), 3 dpe (**F**) and 5 dpe (**G**). Transcripts that exhibited significant differences in abundance (Baggerley’s test; *P* < 0.05) are colour-coded according to their weighted fold change (FC). The expression levels are shown as the mean Log_10_ (TPM + 1) for all of the six biological replicates for both sexes and each age.

**Figure 4:**
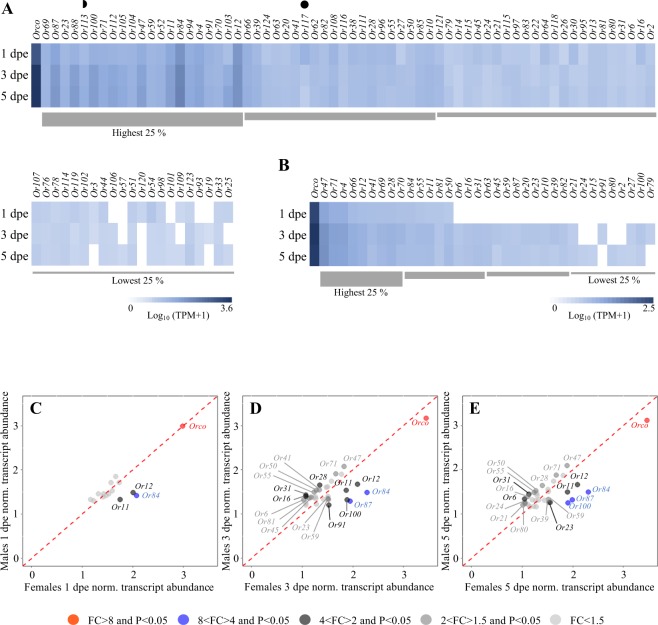
Antennal odorant receptor differential transcript abundance between sexes and across ages in *Aedes aegypti*. Heat plots showing the abundance of odorant receptor (*Or*) transcripts in female (**A**) and male (**B**) *Ae*. *aegypti* at 1, 3 and 5 days post-emergence (dpe). Genes within each gene family are presented left to right from most to least abundant, as determined by the mean abundance of all three ages for each sex. All *Or* transcripts are ordered from left to right from the highest to the lowest quartile, except the co-receptor *Orco*, which is not included in the quartile analysis, and is found at the extreme left. Note differences in scale in (**A**,**B**). Genes differentially abundant between 1 dpe and 5 dpe (◗), and genes differentially abundant between both 1 dpe and 3 dpe, and 1 dpe and 5 dpe (•), were identified using the beta-binomial Baggerley’s test and a false discovery rate (FDR)-corrected p-value (*P*) of <0.05 (n = 6) with a FC ≥2. Scatter plots showing the differential transcript abundance of *Or*s in the antennae of both sexes at 1 dpe (**C**), 3 dpe (**D**) and 5 dpe (**E**). Transcripts that exhibited significant differences in abundance (Baggerley’s test; *P* < 0.05) are colour-coded according to their weighted fold change (FC). The expression levels are shown as the mean Log_10_ (TPM + 1) for all of the six biological replicates for both sexes and each age.

**Figure 5:**
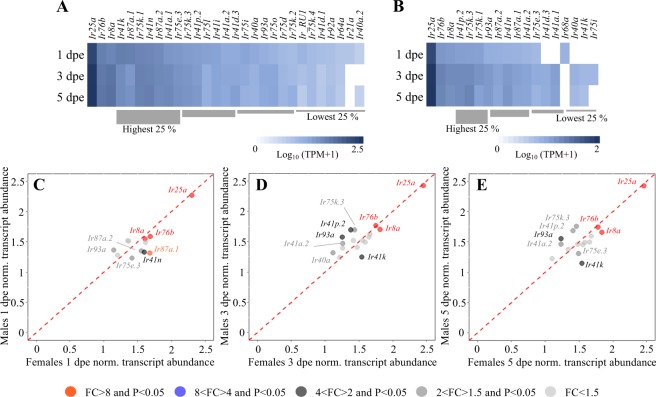
Antennal ionotropic receptor differential transcript abundance between sexes and across ages in *Aedes aegypti*. Heat plots showing the abundance of ionotropic receptor (*Ir*) transcripts in female (**A**) and male (**B**) *Ae*. *aegypti* at 1, 3 and 5 days post-emergence (dpe). Genes within each gene family are presented left to right from most to least abundant as determined by the mean abundance of all three ages for each sex. All *Ir* transcripts are ordered from left to right from the highest to the lowest quartile, except the co-receptors *Ir25a*, *Ir76b* and *Ir8a*, which are not included in the quartile analysis, and are found at the extreme left. Note differences in scale in (**A**,**B**). Scatter plots showing the differential transcript abundance of *Ir*s in the antennae of both sexes at 1 dpe (**C**), 3 dpe (**D**) and 5 dpe (**E**). Transcript that exhibited significant differences in abundance (Baggerley’s test; *P* < 0.05) are colour-coded according to their weighted fold change (FC). The expression levels are shown as the mean Log_10_ (TPM + 1) for all of the six biological replicates for both sexes and each age.

The expression of chemosensory-related genes in *Ae*. *aegypti* is dependent on both sex (Figs 3–5)^7,14^ and age (Figs 3–4)^26^. For both *Ae*. *aegypti* and *An*. *coluzzii*, a sexual dimorphism has been described, with males and females exhibiting few sex-specific, and sexually enhanced, chemosensory genes expressed (Figs 3–5)^7,14,43^. We postulate that the differences observed in patterns of abundance between males and females with age may be linked to the initiation of sexual receptivity and the development of competence to host seek^30,31,39,44^. Here, we present a description of the age- and sex-dependent expression of genes from the chemosensory-related gene families.

### Soluble olfactory proteins - OBPs and CSPs

As in other insects^6,10,11^, OBPs and CSPs were highly detected in the antennae of both male and female *Ae*. *aegypti* (Fig. 3A–D). The members of the OBP gene family exhibited the highest transcript abundances, with the same number (32) of genes identified in both sexes (Fig. 3A,B), which corresponds to previous findings^7^. The OBPs demonstrated the greatest variation in transcript abundance between sexes^7,43^ and across ages (Fig. 3E–G)^7^ among the chemosensory-related genes identified in this study. The majority of the *Obp*s detected, displayed a higher transcript abundance in females compared with males, which supports previous findings in *Ae*. *aegypti*^7^ and *An*. *gambiae*^45,46^. Although, the same number of *Obps* was detected in males and females, three (*Obp19*, *Obp25* and *Obp61*) were detected only in females (Fig. 3A) and another three (*Obp21*, *Obp23* and *Obp26*) only in males (Fig. 3B). Changes in transcript abundance across ages were observed in both sexes, with significant weighted fold changes (FCs) described for select genes between 1 and 3 dpe (Fig. 3A,B), coinciding with the onset of attraction to human odours in both males and females at 3 dpe (Fig. 1b). A similar observation has been shown in females of *Anopheles culicifacies*^47^. The *Obp22* was significantly more abundant in females at 1 dpe, whereas *Obp12*, *Obp13* and *Obp61* were more abundant in 3 dpe females (Fig. 3A). A similar change in abundance was observed for *Obp13* and *Obp22* in males (Fig. 3B). Another gene, *Obp10*, was consistently found to be more abundant in males at all ages (Fig. 3E–G), in line with a previous report^48^. In summary, despite a high number of *Obp* genes detected in the antenna, and at high abundance, only a few genes were identified with significant differential abundant between sexes and across ages.

Until now, only a few OBPs have been functionally characterised in mosquitoes. Of these, direct evidence for function has been described for OBP1, OBP10 and OBP22^49–53^. The *Obp1* was significantly more abundant in females at all ages (Fig. 3E-G), and the protein has been shown to bind to sulcatone^51^ and indole^49^, two volatile compounds implicated in host and oviposition site recognition^37,54^, as well as the *Culex* oviposition pheromone^53^. The OBP22 is binding a number of aromatic compounds, commonly found in floral odours^50,55^. This is interesting as *Obp22* was found to be more abundant in 1 dpe mosquitoes (Fig. 3A,B). RNAi knockdown of both *Obp22* and *Obp10* significantly reduces the probing time in female *Ae*. *aegypti*^52^. The predicted ligands for OBP10, which is more abundant in males (Fig. 3C–E), as well as for OBP12 and OBP13, which are more abundant in 3 dpe mosquitoes (Fig. 3A,B), are ammonia and lactic acid^8^. These compounds have both been described as attractants for mosquitoes^31,56^.

A similar number of CSPs (7) was detected in both sexes, which is in line with that previously reported^7^. Of these, *Csp01957* and *Csp01985* were significantly more abundant in males and females, respectively, at 1 dpe and 3 dpe (Fig. 3C,D). While *Csp01985* continued to be more abundant in 5 dpe females, *Csp05687* was the only gene found to be more abundant in 5 dpe males (Fig. 3D). These results are consistent with previous studies on *Ae*. *aegypti* and *An*. *gambiae*^7,46^. Besides the described sexual dimorphism, three *Csps*, *Csp0591*, *Csp05710* and *Csp05687*, were found to be differentially abundant between 1 dpe and 3 dpe mosquitoes (Fig. 3C,D), suggesting that these genes are involved in the maturation of the olfactory system. The functional role of CSPs in mosquitoes and indeed in other insects remains elusive.

This study demonstrates that the detection and the abundance of a select number of small soluble binding proteins in the antennae of *Ae*. *aegypti* are sex specific and age dependent. The proteins encoded by these genes are favourable candidates for continued functional studies, to assess how these proteins contribute to the survival and fitness of mosquitoes. Future functional characterisation will be required to further investigate the implication of OBPs as components involved in the increase of host attraction in newly-emerged mosquitoes.

### Odorant receptors

In both males and females, *Ors* are one of the most abundant gene families, with the co-receptor *Orco* presenting the highest level of abundance in the antennae of all three ages (Fig. 4A,B), which is in line with previous studies on mosquitoes^7,14,43^. The Or-signalling pathway plays a key role in the detection and discrimination of sugar and blood hosts^36,37^. Both sexes of *Ae*. *aegypti* display similar odour-mediated behaviours once they have achieved sufficient maturation for flight, 12 to 24 h post-emergence^31,39^. Both sexes initially seek sugar sources, followed within 24 to 48 h with the onset of vertebrate host seeking^31,39^. This suggests that at least a subset of the Ors identified in both males and females at 1 dpe are involved in seeking energetic resources, whereas those that are differentially expressed in older mosquitoes are likely involved in driving the odour-mediated host seeking behaviour of both sexes.

Despite normalising *Or* abundance across sexes to account for fewer antennal chemosensory trichoid sensilla in males^40,57,58^, we demonstrated a lower number of Ors to be present in males compared to females at all ages (Fig. 4A,B). While the overall number of *Ors* detected over the first 5 days in females (88) corresponds to that previously reported (88)^7,14^, we demonstrated a substantially lower number of *Ors* present in males (36), compared with the 75^14^ and 95^7^ *Ors* previously reported. Thus, in contradiction with previous studies on older *Ae*. *aegypti*^7^ and *An*. *gambiae*^43^, our results suggest a functional specialisation in the antennae between the sexes in newly-emerged *Ae*. *aegypti*.

In males, only 15 *Or*s, including *Orco*, are detected in 1 dpe individuals, none of which increased in abundance above FC 1.5 as males matured to host seeking (Fig. 4B). Most of these *Or*s (12) had an abundance equivalent to that found in 1 dpe females, indicating a common function of these Or pathways in newly-emerged adults. Only three of these *Or*s (*Or11*, *Or12* and *Or84*) were more abundant in females (Fig. 4C). These three *Or*s were consistently more abundant in females compared to males as the adults aged (Fig. 4D,E). At the onset of host seeking at 3 dpe, three additional *Or*s (*Or81*, *Or87* and *Or100*) were found to be more abundant in females than males (Fig. 4D), and in 5 dpe mosquitoes, *Or23* was found to be significantly more abundant in females (Fig. 5E). In contrast, the abundance of *Or81* was no longer found to be significantly different between the sexes (Fig. 5E). As males matured, three additional *Or*s were found to be differentially abundant in the male antennae (*Or16*, *Or28* and *Or31*). Of these, *Or16*, along with *Or6*, were more abundant at 5 dpe (Fig. 4D,E).

While no male-specific *Or*s were found to be detected in the antennae, 45 *Or*s exhibited female-specific expression (Fig. 4A,B), of which five (*Or26*, *Or27*, *Or97*, *Or103* and *Or123*) had significantly lower abundance in 1 dpe compared with older females (Fig. 4A). Of these, *Or103* is significantly more abundant in the human-preferring strain of *Ae*. *aegypti*^37^. This sex-specific abundance of *Or*s corresponds with the fact that the females, but not the males, exhibited an avoidance to human odours at 1 dpe (Fig. 1B). In addition to differences between the sexes in abundance, recruitment, *i*.*e*. the expression of additional *Or*s in the antennae, was observed in males predominantly at 3 dpe, but also to a lesser extent at 5 dpe (Fig. 4B). A similar increase in recruitment, albeit to a lesser extent, was observed in females (Fig. 4A). While recruitment of *Or*s corresponding with adult age represents a novel finding in mosquito antennae, an increased *Or* abundance over time has been described previously for *Ae*. *aegypti* and *An*. *coluzzii*, correlating with an enhanced behavioural and physiological sensitivity to their cognate ligands^26,35^ [Omondi *et al*., submitted]. Besides the observed increase in abundance, we also noted a significantly lower abundance of one particular gene, *Or117*, in 1 dpe females compared to older females (Fig. 4A). This gene may play a similar role in regulating the onset of host seeking as that proposed for *An*. *coluzzii*, in which the regulation of *AcolOr39* is tentatively linked to the reduced detection of a host repellent compound by the female during sexual maturation (Omondi *et al*., submitted).

Among the *Or*s exhibiting sex- or age-dependent differences in abundance, none have been functionally characterised. However, of the *Or*s found to be reliably detected, OR2, OR4 and OR10 (Fig. 4A,B) have been shown to respond to ecologically relevant volatile compounds. The OR2 and OR10 respond to indolic and phenolic compounds^59^ shown to act as oviposition attractants^54,60^. Although OR4 has been shown to detect sulcatone, a volatile emitted in high levels by humans^37^, it is interesting to note that its gene is not differentially expressed at the onset of host seeking, implicating other *Or*s to be involved in this process. Future functional characterisation of the differentially regulated *Or*s will be required to elucidate the modulation of host seeking in both males and females.

### Ionotropic receptors

Overall, fewer *Irs* were found to be detected in males (18) and females (29) compared to that which has been previously reported in older mosquitoes^7^, with no *Irs* found to be differentially abundant across ages (Fig. 5A,B). The low number of *Irs* and *Ors* detected in maturing males suggests a different rate of canonical olfactory receptor accumulation between sexes. The functional significance of this difference may relate to differences in life history traits, reflecting variation in energetic demands between sexes^61,62^. Despite normalising for the discrepancy in the number of grooved-peg sensilla presumed to house the OSNs expressing these receptors, only *Ir41p*.*2* and *Ir93a*, of the *Irs* present in both sexes, were found to be more abundant in males (Fig. 5C–E). In contrast, *Ir41k*, *Ir87a*.*1* and *Ir41n* were more abundant in females (Fig. 5C–E), which is consistent with previous findings^7^. Amongst the 29 *Irs* identified in females, 12 were female specific (Fig. 5a). Thus, in contradiction with previous findings in older *Ae*. *aegypti*^7^, we hereby propose that both the expression and the rate of accumulation of *Irs* are sex-specific while they are not influenced by age in newly-emerged *Ae*. *aegypti*. The demonstrated differences in the number, presence and abundance of *Irs* suggest a potential difference in the coverage of the odour space detected by this receptor class between males and females.

Of the three tuning Irs that have been functionally characterised in mosquitoes, AgamIR75k, AgamIR41a and AgamIR41c^16^, are homologous with those found to be highly abundant in both sexes of *Ae*. *aegypti* (Fig. 5A,B)^15^. The complex AgamIR75k along with the co-receptor AgamIR8a is activated by carboxylic acids^16,63^, which are major components of human sweat, and are integral for the host-seeking behaviour of most mosquito species^12,64,65^. Functional analysis of AgamIR41a and AgamIR41c along with the two co-receptors AgamIR76b and AgamIR25a demonstrated a specificity for amines^16,63^, another class of semiochemicals involved in host-seeking by adult female mosquitoes^66,67^. The functional characterisation of IR41a and IR75 in both *An*. *coluzzii* and *D*. *melanogaster*, however highlights differences in specificity of homologous receptors^16^. Homologues from the AgamIR75 and AgamIR41 complexes in *Ae*. *aegypti* are differentially abundant between sexes supporting the hypothesis of variation in odour space coverage. Additional functional analysis is required to further elucidate how these and other IRs detect ecologically relevant cues.

### Other membrane-bound proteins (GRs, TRPs, PPKs and SNMPs)

Besides the members of the canonical chemoreceptor families, sex- and age-dependent changes in transcript abundance were observed in other membrane-bound proteins, including GRs, TRPs, PPKs and SNMPs. While these families are not exclusively involved in olfaction, members of each of these proteins have been shown to exhibit chemosensory functions. Of the 27 and 29 *Grs* previously described to be detected in the antennae of male and female *Ae*. *aegypti*, respectively^7^, only three, *Gr2*, *Gr4* and *Gr6*, were present in females, while only one, *Gr6*, was detected in males. This again suggests a delayed accumulation of chemosensory receptor gene expression in mosquitoes during sexual maturation. Transcript abundance of *Gr6* was independent of both sex and age. The functional role of these receptors has not been determined in mosquitoes, however, *Gr4* and *Gr6*, are homologous with sugar receptors described in *D*. *melanogaster*^68^. The gene *Gr2*, a homologue of the CO_2_-sensitive GR, *Gr1*, is widely expressed in chemosensory organs in *Ae*. *aegypti*^69,70^, however its role remains elusive.

Of the 15 and 13 TRPs previously described in the antennae of female and male *Ae*. *aegypti*, respectively^7^, we identified ten and eight genes in this study. Of these genes, three, *nompC*, *nan* and *iav*, were found to be significantly more abundant in males compared to females across all ages tested (FC > 2, *P* < 0.05; Supplementary Files 2–3). Another TRP, TRPA1, has been shown to play a role as a heat-sensor in host-seeking female *An*. *gambiae*^71^ and *Ae*. *aegypti*^72^, and to be required to trigger aversion to citronellal in *D*. *melanogaster*, an insect repellent component^73^. This receptor is of potential interest as it was more abundant in 1 dpe compared to 5 dpe females (2 < FC > 1.5, P < 0.05) and not detected in males. The observed change in abundance may reflect the requirement of young females to detect a specific thermal environment to reduce the time and energy expended for their initial blood meal^74^.

Pickpocket (PPK) channels are members of the family of amiloride-sensitive degenerin/epithelial sodium channels (DEG/ ENaC) that were first described in *D*. *melanogaster*, of which some have been shown to be involved in chemoreception^20^. PPK channel genes are broadly detected across the chemosensory appendages in *Ae*. *aegypti*^7,69,70^, with seven *Ppks* being detected in the antennae among both sexes, five in each sex. Of these, we detected only one, *Ppk00926*, in the antennae of both males and females, and one, *Ppk00873*, exclusively in males. Neither of these PPKs have been functionally characterised in mosquitoes, however, *ppk00926* is related to the PPK subfamily V, which are predominantly mechanosensory^20^. While none of the *Ppk*s displayed an age-dependent change in abundance, *Ppk00926* was significantly more abundant in males (FC > 2, P < 0.05) in comparison to females^7^, which is intriguing as other PPKs have been shown to be involved in courtship behaviour in male *D*. *melanogaster*^75^.

Of the two identified SNMPs in *Ae*. *aegypti*^24,70^, both were detected in male and female antennae of all three ages. *Snmp1* was the only gene that was differentially abundant (FC > 2, P < 0.05), being more abundant in females than males at all ages. Neither of the *Snmp*s demonstrated age-dependent changes in abundance. While the functional role of SNMPs is still unknown, members of this membrane-bound protein family have been shown to play a role in pheromone detection in other insects^23,76^.

In summary, of all the genes belonging to the olfactory-related membrane-bound proteins, only two genes, *SRCB3* and *TrpA1*, exhibited age-dependence, being more abundant in 1 dpe than older mosquitoes (2 < FC > 1.5, P < 0.05). This suggests that there is a striking difference in the regulation of these non-canonical olfactory genes compared with their canonical counterparts, with respect to sexual maturation.

## Conclusion

Both male and female *Ae*. *aegypti* alter their behavioural response to human odour during sexual maturation. This behavioural development is correlated with an overall change in transcript abundance and, more specifically, age-dependant changes in select canonical chemosensory genes, occurring mainly between 1 and 3 dpe. These genes provide novel targets for functional characterisation, which may in turn lead to the development of tools and strategies against vector borne diseases.

## Methods

### Mosquito rearing

*Aedes aegypti* (Rockefeller strain) were reared as previously described^77^. Following the adult emergence, the mosquitoes were separated according to sex within 24 h, prior to sexual maturity^78^. All mosquitoes were provided *ad libitum* access to 10% sucrose. Mosquitoes were starved with access to water for 6 h prior to both the behavioural assay and the tissue collection. In order to reduce the effect of the circadian rhythm on gene expression, the behavioural analysis and tissue collections were performed between zeitgeber time 8–11, corresponding to the peak of host seeking, commonly observed in the late photophase and early scotophase^79^.

### Mosquito behaviour

Host seeking was investigated in a Plexiglass® Y-tube olfactometer (Fig. 1A)^80^, illuminated from above at 500 lx. A charcoal-filtered and humidified air stream (25 ± 2 °C, 65 ± 2% relative humidity) was passed through the olfactometer at 30 cm s^−1^. Mosquitoes were transferred into meshed release cages and acclimatised in the behavioural room for 2 h prior to the experiments. Two minutes prior to analysis, the release cage was positioned in the Y-tube olfactometer in order to give the insect time to acclimatise to the air current present in the olfactometer. Mosquitoes were transferred and released individually, and given the choice between an odour stimulus generated by a sock worn for 7 h prior to the assays and a sock cleaned with odourless soap (Dove, Mannheim, Germany), inserted upwind of the capture cages (Fig. 1A) prior to the beginning of the experiment. Mosquitoes were given 3 min to make a choice between the two arms of the olfactometer. Mosquitoes that did not make a choice and stayed either in the release cage or in the central arm were recorded as “no-choice”. In order to prevent bias within the olfactometer, the two arms were inverted every three mosquitoes. Moreover, control tests, in which both chambers contained worn socks, were conducted every day (data not shown). The attraction to human odour was scored as a preference index generated by (T − C)/(T + C) where T is the proportion of mosquitoes entering the arm with human odour, while C is the proportion of mosquitoes entering the control arm. The behavioural response to human odours and its dependence on age and sex were analysed by using R software (version 3.5.0)^81^ with a binomial generalised linear model (GLM) followed by Tukey’s multiple comparisons *post-hoc* test.

### Tissue dissection and RNA extraction

Antennae of adult females and males, 1, 3 and 5 dpe, were collected between zeitgeber time 8–11. In order to minimise variation, the antennal tissue was collected from individuals of matched cohorts for all three ages. The antennae were removed from cold-anesthetized individuals using forceps and immediately transferred into RNAlater® (Thermo Fisher Scientific, Sweden), stored at room temperature overnight, and then transferred to −80 °C until RNA extraction. A total of six independent biological replicates were generated for each experimental group, each containing 150 pairs of antennae. The tissues were disrupted and homogenised using a power pestle with a disposable RNAse free plastic pestle (VWR International, United Kingdom). Total RNA extraction and DNAse digestion were performed using the RNeasy Mini Kit (Qiagen, Sweden) following the manufacturer’s protocol. Total RNA samples were stored at −80 °C. Unfrozen aliquots of RNA were assessed for quantity through the Qubit Quantification RNA assay (Qubit, Life Technologies, Sweden), and quality using the Experion™ RNA StdSens Analysis kits (BIO-RAD, USA), on an Agilent 2100 Bioanalyzer (Agilent Technologies, Waldbronn, Germany).

### Sequencing, read mapping and gene annotations

Total RNA samples were shipped on dry ice to BGI Tech Solutions (Hong Kong, China) for complementary deoxyribonucleic acid (cDNA) library construction and RNA-Seq quantification library (Illumina HiSeq™ 2000). Library construction of cDNA was realised using a BGI proprietary protocol using DNA polymerase I and deoxyribonucleotide triphosphate (dNTPs), following RNase H treatment. Prior to the quantitative analysis, strict quality controls were performed according to BGI’s standards. Following sequencing, the adaptor sequences were removed from the raw reads, and reads containing a high proportion (>10%) of unknown bases and low-quality reads (minimum quality score of 20) were removed using the software package RSEM^82^. Quantitative single-end sequencing of antennal RNA from each of the 36 samples generated a mapping of over 22 million cleaned reads per library. Clean reads were mapped to the reference genome (AaegL3), obtained from ENSEMBL^83^, which was annotated using an updated version of the transcriptome AaegL.RU^7^, with additional manual curation of 44 chemosensory-related transcripts (Additional File 4), with CLC Genomics Workbench 10.0.1^84^.

### RNA-Seq analysis and differential expression analysis

Quantile-normalisation was performed on each library in order to limit bias due to the presence of highly expressed genes, and to permit the detection of transcripts with low expression levels. Genes with a read abundance below the threshold of 1 RPKM, commonly used to reduce noise^7^, were identified and removed from subsequent analysis. Genes detected above this background level of abundance were considered to be reliably detected. In order to investigate the RNA expression in the antennae of both male and female *Ae*. *aegypti* adults, we report and compare the transcript abundance in units of transcript per kilobase million (TPM) between the sexes at 1, 3 and 5 dpe; and among these time points for each sex separately. From the whole transcriptome dataset, (Additional Files 2–3), we identify candidate genes-of-interest according to their differential FC in transcript abundance, as assessed using the beta-binomial Baggerley’s test^85^ and a false discovery rate (FDR)-corrected p-value (*P*) of <0.05 (n = 6)^86^. Genes were considered as differentially expressed at a FC ≥ 2 and of potential interest if the genes exhibited 1.5 ≤ FC < 2, both with *P* < 0.05.

To compensate for the reduced number of sensilla on the male antennae, factors of 2.9 and 3 were applied to the male transcript abundance of ORs and IRs, respectively. These factors are based on the ratio of the sensilla likely to express these receptors, the trichoid (532/175) and the grooved-peg (105/36) sensilla, respectively, on the antenna of female and male *Ae*. *aegypti*^40,57,58^.

## Electronic supplementary material

Supplementary data legends

Additional file 1

Additional file 2

Additional file 3

Additional file 4

## Data Availability

All cleaned reads from each of the six libraries (SAMN10497039-SAMN10497044) are deposited in the NCBI traces database as SRA study SRP171130####### under the BioProject number PRJNA507369. The data will be released in the database upon acceptance of the manuscript. Gene set annotations, expression data and sequences of modified genes are available as additional files with this manuscript.
