# Supplementary material for: Sex and age modulate antennal chemosensory-related genes linked to the onset of host seeking in the yellow-fever mosquito, Aedes aegypti
Source: Sci Rep. 2019 Jan 10;9:43. doi: 10.1038/s41598-018-36550-6 (PMC6328577; doi:10.1038/s41598-018-36550-6)
Supplement: Supplementary file 1 — Supplementary data legends [file 41598_2018_36550_MOESM1_ESM.docx]

**Sex and age modulate antennal chemosensory-related genes linked to the onset of host seeking in the yellow-fever mosquito, *Aedes aegypti***

Anaïs Karine Tallon^1^, Sharon Rose Hill^1§^, Rickard Ignell^1§*^

^1^Disease Vector Group, Department of Plant Protection Biology, Swedish

University of Agricultural Sciences, Alnarp, Sweden

[Anais.tallon@slu.se](mailto:Anais.tallon@slu.se), [Sharon.hill@slu.se](mailto:Sharon.hill@slu.se), [Rickard.ignell@slu.se](mailto:Rickard.ignell@slu.se)

^§^Shared last authors

*Corresponding author: Rickard.Ignell@slu.se

**Supplementary information**

**Additional file 1**: Lists the total number of reads, mapped reads (%), transcripts and reliably detected transcripts (threshold > 1 RPKM) for the transcriptomes of each of the 36 biological replicates (XLSX 11.4 kb).

**Additional file 2**: The transcript abundance (in TPM) for the six biological replicate transcriptomes of female *Aedes aegypti* antennae at 1, 3 and 5 day(s) post-emergence (XLSX 2.2 Mb).

**Additional file 3**: The transcript abundance (in TPM) for the six biological replicate transcriptomes of male *Aedes aegypti* antennae at 1, 3 and 5 day(s) post-emergence (XLSX 1.7 Mb).

**Additional file 4**: Lists the 44 additional chemosensory-related transcripts used to update the annotated version of the transcriptome AaegL.RU ^7^ (XLSX 42 kb).
